# Commencement of Buffalo Medical College

**Published:** 1886-03

**Authors:** 


### Commencement of Buffalo Medical College.

The annual meeting of the Alumni and the commencement exercises of the Buffalo Medical College were held last Tuesday.

The Alumnal Association met at 10 A. M. There was a fair attendance.

In the absence of President Dewey and First Vice-President Diehl, Dr. D. W. Harrington, Second Vice President, took the chair. Dr. John J. Walsh was in his position as Secretary, and Dr. E. C. W. O'Brien, in his capacity of Treasurer, was busy with the receipt of dues.

Dr. Park made an address of welcome. He announced that there had been added to the faculty, Dr. Hopkins, who accepts the chair of diseases of the nervous system; Dr. Hinkel, the chair of laryngology; Dr. Vandenburgh was appointed Adjunct Professor of Chemistry; Dr. Bartow, Professor of Orthopedic Surgery.

The nominating committee, Drs. Gregory, Barker and Phelps, reported the following ticket for the ensuing year, which was declared duly elected:

*President*, C. Diehl; *First Vice-President*, Henry Lapp, Clarence; *Second Vice-President*, D. W. Harrington; *Third Vice-President*, C. S. Pugsley, Oakfield; *Fourth Vice-President*, Charles A. Ring; *Fifth Vice-President*, S. I. Wells, Smethport, Pa.; *Secretary*, John J. Walsh; *Treasurer*, E. C. W. O'Brien; *Trustees*,

W. C. Phelps, P. W. VanPeyma, Henry Lapp, E. C. W. O'Brien, Horace Hoyt, Aurora; *Executive Committee*, James W. Putnam, W. H. Thornton, W. G. Gregory; Charles Cary, *ex-officio* Member of the Faculty; C. Diehl, President of the Association.

The morning session then adjourned.

In the afternoon, the Alumni listened to three excellent papers, "The Legal Status of Medical Practitioners," by Ansley Wilcox, Esq.; "Diseases of the Period of Dentition," by Dr. W. C. Barrett; "Relations of Sewers and Sewage to Cities," by G. E. Mann, C. E. This paper was discussed by Dr. Andrews in a most intelligent manner, also by Drs. Briggs and Putnam.

At a meeting of the Council, the following received the degree of Doctor of Medicine:

Frederick A. Strasenburgh, Lima, N. Y.; Dennis J. Constantine, Black Rock, N. Y.; Charles George Steele, Buffalo, N. Y.; George H. Westinghouse, Buffalo, N. Y.; William John Brand, Buffalo, N. Y.; Albert E. Campbell, Sunny Brae, N. S.; Walter M. Litchfield, Franklinville, N. Y.; Eugene H. D. Ringueberg, Lockport, N. Y.; Louis Pfandhoefer, Buffalo, N. Y.; George Hoffman Penrose, Butte, Mont.; William A. Hoddick, Buffalo, N. Y.; Charles A. Boysen, Buffalo, N. Y.; George Edward Smith, Alexander, N. Y.; Robert R. Seyse, Java Village, N. Y.; Myron Lucius Cooley, New Haven, Ct.; John David Davis, Cassadaga, N. Y.; Mr. Onontiyoh, Tuscarora, N. Y.; Alonzo L. Richman, East Kendall, N. Y.; Charles Bell Signer, Buffalo, N. Y.; George Sherman Palmer, Buffalo, N. Y.; Myron Henry Parkhill, Hornellsville, N. Y.; John J. Krim, Buffalo, N. Y.; George Gratton Jones, S. Dansville, N. Y.; Fred. H. Ehinger, Alden, N. Y.; Elmer D. Williams, Eddyville, N. Y.; Michael Retel, Buffalo, N. Y.; Edward Thomas Stevens, Buffalo, N. Y.; Willard P. Rivenburgh, Ghent, N. Y.; Gustav A. Pohl, Mainz, Germany; William Turck, Orleans, N. Y.; Charles Peter Eller, Buffalo, N. Y.; George William Lane, Beaver Dam, N. Y.; William W. Hadley, Black River, N. Y.; Carl Wm. Auel, Wassmuthshausen, Germany; John George Meidenbauer, Buffalo,

N. Y.; Burton Edgar Manchester, Hartland, N. Y.; Louise Downer, Peterboro, N. Y.; John F. Carleton, Rochester, N. Y.; Nicholas McCabe, Batavia, N. Y.; Stoughton Rowley Wheeler, E. Bloomfield, N. Y.; Henri Michel Auger, Raxton Pond, Canada; James Louis Meek, Alton, Canada; James Sanderson Wells, Fort Erie, Canada.

In the evening, the commencement exercises were held in Liedertafel Hall, and the diplomas were given to each graduate as conferred by the Council. The names are given above. Dr. Adams, President of Cornell University, gave an interesting address to the class; and Rev. Herbert C. Lord addressed the Alumni, he was truly eloquent, and was interrupted from time to time with bursts of applause.

The annual banquet of the Alumnal Association was held at the Tifft House in the evening. About 300 gentlemen and one lady sat down to table. Dr. Judson B. Andrews was toast master. The following toasts were responded to:

Our Alma Mater—W. W. Potter, M. D.

Our Deceased Alumni and Associates.

The Medical Profession—Simeon T. Clark, M. D.

Our Sister University—Prof. Charles K. Adams.

Faculty of the University—Dr. Roswell Park.

The Clergy—The Rev. Mr. Fuller.

The Legal Profession—Porter Norton, Esq.

The Press—Isaac Bromley.

Sanitary Medicine—A. H. Briggs, M. D.

The Graduating Class—Dr. W. M. Litchfield.

Dr. Andrews presided in his usual dignified and happy manner. Under his guidance the affair proved a great success. The doctor always goes in to make a success of everything he undertakes, and this proved no exception to the rule. The toasts were responded to as above. The speech of Dr. Clark, of Medical Department of Niagara University, was an effort well worthy of the Doctor, and he maintained his reputation as an orator. Mr. Isaac Bromley responded for the press in his usual happy manner. The address of Dr. Litchfield, of the graduating class was well written and highly favorable to his associates, the doctor predicts this will be the banner class of the school. Thus ended an enjoyable and profitable day.

